# The Influence of Diseases of the Air-Passages on the Teeth

**Published:** 1903-09

**Authors:** A. G. Minshall

**Affiliations:** Northampton, Mass.


### THE INFLUENCE OF DISEASES OF THE AIR-PASSAGES ON THE TEETH.[1]

BY A. G. MINSHALL, M.D., NORTHAMPTON, MASS.

Mr. Chairman and Gentlemen,—The time has undoubtedly arrived when it is appreciated by all that dental science stands in such close relationship to the other branches of the great art of scientific medicine that the work of the dentist and that of the physician have innumerable points of contact and common interest to both professions. The American Medical Association for some sixteen years has had a Section on Oral and Dental Surgery and Stomatology, under the efficient guidance of Dr. E. S. Talbot, of Chicago, and at the last meeting of that body it was recognized and approved that reputable members of your profession shall be eligible for associate membership in the Association, to the mutual benefit of us all. These facts enable me the more readily to accept the invitation to address you on one of the many problems which confront us in common in our daily experience.

You all have frequently been called upon to rectify the deformities of the teeth, and doubtless have studied the causation of these defects. I propose to briefly consider some of these causes as they appear to those of us interested in diseases of the upper air-passages.

By far the most important and common of these factors is nasal obstruction and consequent mouth-breathing, and the age at

---

[1] Read before the Massachusetts Dental Society Meeting, held June 3 and 4, 1903, Boston, Mass.

which it is most apt to appear is just previous to the eruption of the permanent teeth. There may be obstruction in any part of the air-passages leading from the nostrils to the beginning of the œsophagus, but the condition most common at the period named is that known as adenoids, an overgrowth of the lymphoid tissue normally present at the upper part of the pharynx, the so-called pharyngeal tonsil; with or without this enlargement there may be a similar hypertrophy of the faucial or true tonsils. Less commonly in children, but more so in adults, we meet with the spongy swelling of the nasal mucous membranes, either general or localized, in the form of polypoid excrescences. The chief interest to the dentist lies, of course, in the results produced by these conditions during the periods of life when the jaw and teeth are undergoing their development and growth. In studying the development of the bones which enter into the formation of the hard palate in particular, we find that although the ossification of the upper jaw is not thoroughly understood, it is an undoubted fact that the palatal process, with the alveolus of the four incisors, ossifies quite late in comparison to the remainder, and in consequence these teeth are the most frequently and severely displaced.

If a child is a mouth-breather from any reason, jaw and tooth deformities are likely to arise from several causes: first, it has been demonstrated experimentally and otherwise that there is a general arrest of the proper growth in all the facial bones, and especially in those forming the walls of the nasal chambers; young animals with one nostril plugged present marked atrophy, or, rather, deficient development of the structures on that side; secondly, deformity is brought about by the yielding of the yet plastic palatal arch to the pressure of the air forcibly inspired through the mouth. If the mouth-breathing is only present before the eruption of the permanent teeth, the palate may become dome-shaped, leading to an approximation of the alveolar borders, so as to form an elliptical curve; the whole growth of the jaw will be more or less retarded, but the teeth will be in normal position if nasal respiration is restored. If, however, the cause continues during the second dentition, the palate becomes even more elevated, forming a Gothic arch, the anterior part of the alveolus inclines forward, and the jaw assumes a V-shape. These changes produce the following alterations in the position of the teeth: the central incisors are rotated so that their lingual surfaces look towards one another and are tilted forward, while the lateral incisors and frequently the premolars are pushed inward, the molars turning outward. As the upper and lower jaws do not develop at the same rate, dental articulation becomes altogether interfered with.

In studying the actual physical cause of these malpositions, we find that the prime factor in the case is the upward extension of the palatal arch, which brings the sides together, so that, as the regular number of teeth begin to demand room, they can only find it forward. The molars seem to suffer least, possibly because they fulfil the most important function of grinding. The incisors and canines suffer most because there is nothing to prevent them from being forced out in front by the lateral pressure.

I have brought no specimens to exhibit, knowing that every dental office contains casts which show all these conditions, but I do not think that there is so general an appreciation of them by your profession as there should be in reference to a large field of your work,—the straightening of the front teeth in children, which it is useless to attempt with success so long as the cause of the malformation is still active. Not only does mouth-breathing affect the position of the teeth, but it impairs their nutrition by its bad effects on the secretions of the mouth and the digestion in general, leading to premature softening and caries.

Let us now briefly consider the causes and results of mouth-breathing after puberty. In the great majority of cases this will be found to be due to swelling of the mucous membrane lining the nasal passages, and to the secondary deformities which have been caused by the presence of adenoids at an earlier period, most commonly a deflection of the septum which has been distorted by the upward pressure of the excessively arched palate. From this time onward we cannot, of course, expect to do much in the way of restoring the normal shape of the jaw, but we can, by restoring nasal respiration, improve the general aspect of the face and the condition of tooth vitality, both by rendering the oral cavity less septic and by raising the tone of the general health. So much for the effect of mouth-breathing. There are some other conditions which may cause the dentist trouble in which the fault arises from the nasal passages, particularly some obstinate neuralgias arising from pressure on the nerve-endings within the nose from new growths, enlarged turbinated bones, and similar sources of irritation. It is advisable that the dentist should be on the lookout for these difficulties, so that he may save himself much fruitless and tedious work, and I will briefly run over a few points of value for diagnosis. First, as to children with adenoids. You may or may not have mouth-breathing when you see the case in your office, but the high-arched palate, the broadened bridge to the nose, leading to the appearance known as "frog-face," and a catarrhal discharge from the nostrils are common signs; in other cases you will see the well-marked mouth-breather, with the "rabbit-mouth," upper lip projected forward, and jaw dropped, a stupid expression of face, and sometimes a dulness of hearing. In older people you may suspect nasal irritation from a discharge from the passages, with redness or soreness around the nostrils, a nervous sniffing at intervals, and a tendency towards weakness and redness of the eyes.

I hope that these few hints may be of service to your practice; on many other occasions, when I have come into somewhat intimate contact with members of your profession, I found myself under certain conditions of more or less restraint, not to say actual discomfort. I am thankful to say that this time I feel more at my ease, so much so that I propose to close my mouth when I am ready so to do, as is now the case.

